# Supplementary material for: Coarse Woody Debris Decomposition Assessment Tool: Model validation and application
Source: PLoS One. 2021 Jul 9;16(7):e0254408. doi: 10.1371/journal.pone.0254408 (PMC8270427; doi:10.1371/journal.pone.0254408)
Supplement: Attachment — Submitted filename: Response_To_Editor_Reviewers-MS2.docx [file pone.0254408.s002.docx]

Journal Requirements:

When submitting your revision, we need you to address these additional requirements.

 1. Please ensure that your manuscript meets PLOS ONE's style requirements, including those for file naming. The PLOS ONE style templates can be found at

<https://journals.plos.org/plosone/s/file?id=wjVg/PLOSOne_formatting_sample_main_body.pdf> and

https://journals.plos.org/plosone/s/file?id=ba62/PLOSOne_formatting_sample_title_authors_affiliations.pdf

**Response: Thank you. While revising we have taken care to ensure that PLOS ONE’s style guidelines have been followed. Please let us now if you notice any non-compliance that still needs correction.**

2. In your Methods section, please provide additional information regarding the permits you obtained to collect samples for the present study. Please ensure you have included the full name of the authority that approved the field site access and, if no permits were required, a brief statement explaining why.

**Response:** **Thank you. We added a paragraph to the Study Site to clarify (lines 115 to 122 of the revised manuscript).**

3. Thank you for stating the following in the Acknowledgments Section of your manuscript:

"Funding to install the FWDE was provided by the USDA Forest Service and Michigan
Technological Univ. The model development was supported by the US Dept. of Energy (DE352
SC0016235), US NSF (DEB 1754603) and the USDA Forest Service."

We note that you have provided funding information that is not currently declared in your Funding Statement. However, funding information should not appear in the Acknowledgments section or other areas of your manuscript. We will only publish funding information present in the Funding Statement section of the online submission form.

Please remove any funding-related text from the manuscript and let us know how you would like to update your Funding Statement. Currently, your Funding Statement reads as follows:

"Please see Acknowledgments "

Please include your amended statements within your cover letter; we will change the online submission form on your behalf.

**Response:** **We deleted the words related to funding in the Acknowledgement and will provide the funding information to the online form. We have included the correct funding information in our cover letter, please help us fix the errors in funding information.**

**The correct funding information is as follows:**

**Funder Name: U.S. Department of Energy (DOE, USA)**

**Grant Number: DE-SC0016235**

**Grant Recipient: Dr Carl C Trettin**

**Funder Name: National Science Foundation (NSF, USA)**

**Grant Number: DEB 1754603**

**Grant Recipient: Dr Andrew J Burton**

**Funder Name: Natural Science Foundation (NSF, USA)**

**Grant Number: DEB 1754616**

**Grant Recipient: Dr Jonathan S Schilling**

4. Thank you for stating the following in your Competing Interests section:

"NO"

Please complete your Competing Interests on the online submission form to state any Competing Interests. If you have no competing interests, please state "The authors have declared that no competing interests exist.", as detailed online in our guide for authors at http://journals.plos.org/plosone/s/submit-now

 This information should be included in your cover letter; we will change the online submission form on your behalf.

Please know it is PLOS ONE policy for corresponding authors to declare, on behalf of all authors, all potential competing interests for the purposes of transparency. PLOS defines a competing interest as anything that interferes with, or could reasonably be perceived as interfering with, the full and objective presentation, peer review, editorial decision-making, or publication of research or non-research articles submitted to one of the journals. Competing interests can be financial or non-financial, professional, or personal. Competing interests can arise in relationship to an organization or another person. Please follow this link to our website for more details on competing interests: http://journals.plos.org/plosone/s/competing-interests

**Response: The authors have declared that no competing interests exist.**

5. We note that you have stated that you will provide repository information for your data at acceptance. Should your manuscript be accepted for publication, we will hold it until you provide the relevant accession numbers or DOIs necessary to access your data. If you wish to make changes to your Data Availability statement, please describe these changes in your cover letter and we will update your Data Availability statement to reflect the information you provide.

**Response:** **Data used for the model calibration and validation have been provided in Table 1 and Table 2 and S1 Table, but the missing climate data have to download from Daymet database (Thornton et al., 2016) using the coordinates in Table 1. Users can parameterize the model using the daily minimum and maximum temperature and daily precipitation from Daymet and S1 Table, and soil and land cover information in Table 1, and wood properties in Table 2 for the comparison of the model outputs and the results from the model calibration and validation showed in this paper. Users can parameterize the model for assessing CWD decomposition on WS80 using the related ambient conditions (Table 1 and S1 Table) and CWD in Table 3 to compare the model outputs to the results from model application showed in this manuscript.**

6. PLOS requires an ORCID iD for the corresponding author in Editorial Manager on papers submitted after December 6th, 2016. Please ensure that you have an ORCID iD and that it is validated in Editorial Manager. To do this, go to ‘Update my Information’ (in the upper left-hand corner of the main menu), and click on the Fetch/Validate link next to the ORCID field. This will take you to the ORCID site and allow you to create a new iD or authenticate a pre-existing iD in Editorial Manager. Please see the following video for instructions on linking an ORCID iD to your Editorial Manager account: <https://www.youtube.com/watch?v=_xcclfuvtxQ>

**Response: My ORCID is 0000-0002-0941-8345 and it has been validated in Editorial Manager.**

7. We note that Figures 1 and 2 in your submission contain map images which may be copyrighted. All PLOS content is published under the Creative Commons Attribution License (CC BY 4.0), which means that the manuscript, images, and Supporting Information files will be freely available online, and any third party is permitted to access, download, copy, distribute, and use these materials in any way, even commercially, with proper attribution. For these reasons, we cannot publish previously copyrighted maps or satellite images created using proprietary data, such as Google software (Google Maps, Street View, and Earth). For more information, see our copyright guidelines: <http://journals.plos.org/plosone/s/licenses-and-copyright>.

 We require you to either (1) present written permission from the copyright holder to publish these figures specifically under the CC BY 4.0 license, or (2) remove the figures from your submission:

 7.1.    You may seek permission from the original copyright holder of Figures 1 and 2 to publish the content specifically under the CC BY 4.0 license.

We recommend that you contact the original copyright holder with the Content Permission Form (<http://journals.plos.org/plosone/s/file?id=7c09/content-permission-form.pdf>) and the following text:

“I request permission for the open-access journal PLOS ONE to publish XXX under the Creative Commons Attribution License (CCAL) CC BY 4.0 (<http://creativecommons.org/licenses/by/4.0/>). Please be aware that this license allows unrestricted use and distribution, even commercially, by third parties. Please reply and provide explicit written permission to publish XXX under a CC BY license and complete the attached form.”

 Please upload the completed Content Permission Form or other proof of granted permissions as an "Other" file with your submission.

 In the figure caption of the copyrighted figure, please include the following text: “Reprinted from [ref] under a CC BY license, with permission from [name of publisher], original copyright [original copyright year].”

 7.2.    If you are unable to obtain permission from the original copyright holder to publish these figures under the CC BY 4.0 license or if the copyright holder’s requirements are incompatible with the CC BY 4.0 license, please either i) remove the figure or ii) supply a replacement figure that complies with the CC BY 4.0 license. Please check copyright information on all replacement figures and update the figure caption with source information. If applicable, please specify in the figure caption text when a figure is similar but not identical to the original image and is therefore for illustrative purposes only.

The following resources for replacing copyrighted map figures may be helpful:

 USGS National Map Viewer (public domain): <http://viewer.nationalmap.gov/viewer/>

The Gateway to Astronaut Photography of Earth (public domain): <http://eol.jsc.nasa.gov/sseop/clickmap/>

Maps at the CIA (public domain): <https://www.cia.gov/library/publications/the-world-factbook/index.html> and <https://www.cia.gov/library/publications/cia-maps-publications/index.html>

NASA Earth Observatory (public domain): <http://earthobservatory.nasa.gov/>

Landsat: <http://landsat.visibleearth.nasa.gov/>

USGS EROS (Earth Resources Observatory and Science (EROS) Center) (public domain): [http://eros.usgs.gov/#](http://eros.usgs.gov/)

Natural Earth (public domain): <http://www.naturalearthdata.com/>

**Response:** **We recreated the Figure 1 using the map layer from the public domain Natural Earth (please the caption of Fig.1) as you suggested.**

**We deleted Figure 2 and added some text to Study Site section to describe where our experimental forest (Santee EF) is (lines 106 to 114 of revised manuscript), although the Figure 2 was from public domain, the Center for Forested Wetlands Research, USDA Forest Service.**

[Note: HTML markup is below. Please do not edit.]

Reviewers' comments:

Reviewer's Responses to Questions

**Comments to the Author**

1. Is the manuscript technically sound, and do the data support the conclusions?

The manuscript must describe a technically sound piece of scientific research with data that supports the conclusions. Experiments must have been conducted rigorously, with appropriate controls, replication, and sample sizes. The conclusions must be drawn appropriately based on the data presented.

Reviewer #1: Partly

**Response: Thank you. We have worked to improve the quality of this manuscript based on the comments of the reviewer.**

2. Has the statistical analysis been performed appropriately and rigorously?

Reviewer #1: Yes

**Response: Thanks.**

3. Have the authors made all data underlying the findings in their manuscript fully available?

The [PLOS Data policy](http://www.plosone.org/static/policies.action#sharing) requires authors to make all data underlying the findings described in their manuscript fully available without restriction, with rare exception (please refer to the Data Availability Statement in the manuscript PDF file). The data should be provided as part of the manuscript or its supporting information, or deposited to a public repository. For example, in addition to summary statistics, the data points behind means, medians and variance measures should be available. If there are restrictions on publicly sharing data—e.g. participant privacy or use of data from a third party—those must be specified.

Reviewer #1: Yes

**Response: Thanks.**

4. Is the manuscript presented in an intelligible fashion and written in standard English?

PLOS ONE does not copyedit accepted manuscripts, so the language in submitted articles must be clear, correct, and unambiguous. Any typographical or grammatical errors should be corrected at revision, so please note any specific errors here.

Reviewer #1: Yes

**Response: Thanks.**

5. Review Comments to the Author

Please use the space provided to explain your answers to the questions above. You may also include additional comments for the author, including concerns about dual publication, research ethics, or publication ethics. (Please upload your review as an attachment if it exceeds 20,000 characters)

Reviewer #1: General comments
I found many positive aspects to this manuscript. We absolutely need to be able to predict how dead wood pools respond to climate change and management. We need to move to mechanistic models. We need broad-scale experiments to calibrate and test these models. The current manuscript describes a study in which this has been done and while it is not the end of this process it certainly provides an interesting and very good example of what could be done.
However, the authors seem to gloss over the shortcomings of their work. Don’t get me wrong, for the resources available and the state of the science in this field they did a very nice job. But decomposition of woody debris can take decades to centuries, so a 6 year study is short even for the tropics. Having data at one point in time is better than no data, but it does place some real limitations on the kinds of calibration and predictions possible. Although their experiment and sites spanned a wide range of conditions, they are somewhat limited to those in which woody debris is decomposing within. Again, not the fault of the authors to study as much as they could give resources, etc. But it needs to be pointed out that colder and warmer sites exist globally as well as drier and wetter ones. They largely worked under forest canopies, but after severe disturbances the dead wood might be decomposing in open settings that their experiment did not consider. They tested three species belonging to widespread genera; however, none of these species had strong decay resistance. The experiment likely under estimated the range of decomposition possible within a site. So while I understand that one can only do so much, it would be useful to acknowledge this and then suggest how these limitations might be overcome. Or which ones should be given priority. This would form the basis of a very useful discussion.

**Response: Very nice suggestions. CWD decomposition needs a long time period to complete indeed, decades, and in some cases even more than hundred years. Tree species in our experiment are not enough to encompass the full variety of species, wood density and size that can impact wood decomposition rate. FACE wood was used as it has a unique isotopic signature that lets us track wood C into soil pools. Ultimately, this will let us test other model functions as the soil data becomes available. Because FACE wood is one-time results of the discontinutation of FACE experiments in forests, there was a limited total supply and only a few species available. We encompassed as wide a range of environment as we could, given the amount of FACE wood available. We acknowledge that there are locations both warmer and colder than those we studied and a much wider range in wood size and inherent decay resistance than our study with only three species was able to address. Future testing of the model with experimental results from a wider variety of species, wood sizes and climates is needed to help test and revise model calibration and ensure wider applicability of the model. Where possible, real data on DOC production and movement of wood C into soil pools should be included in such efforts. Text to this effect has been added to Discussion section (see lines 294 to 316 and lines 369 to 378 of the revised manuscript).**

**With regard to canopy gaps, in original text (lines 149 to 152 before revision), we mentioned leaf area index initialization to reflect the degree to which FACE logs were placed under canopy or in more open areas, but we did not give LAI values for all sites. In order to be clear, we revised the sentence (lines 101 to 103 the revised manuscript) and added a sentence to Study Site section (lines 104 to 105 of the revised manuscript), i.e., “**, **leaf area index (LAI) ranges from 0.1 to 5.5 m^2^ m^-2^ (Table 1), with an average of 2.6 m^2^ m**^-2^**” and also added a column “LAI” to Table 1 to show the LAI for each site.**

There are various results in the manuscript that don’t seem to have any methods. I realize that the emphasis is on the model. However, there are many detailed results such as the increase in soil stores of C from CWD decomposition that don’t seem to have any specific methods. Same as for the proportion of decomposition attributed to different causes. All very interesting, but unclear as to how the numbers were derived.
Oddly there is not very much regarding the decomposition rates themselves. It might be useful to start the results section not with model calibration, but with a quick review of the decomposition results. I understand the main focus is on the model, but the calibration and validation do depend on the underlying decomposition data.
I found the discussion to largely be either a rehash of the results or to present what seems to be new results. There is very little actual discussion. In the conclusions there is a sentence or two on the need for more data. That is good and obviously true. But in the discussion the kinds of data or studies could be developed so that this statement has some basis. For example, are longer studies needed? Or more species or places or conditions? Is there a need to improve process level studies to provide better insights into the actual mechanisms? Addressing these issues might lead to a more interesting and useful discussion.

**Response: Sorry for the missing. Some methodologies are not described in great detail in this manuscript because main methods for the model are provided in the first paper of the paired papers, i.e., the first paper of the paired papers is mainly used to describe the detailed methods and the second (this one) is mainly used to show the results from model calibration and validation, and model application. This is why we requested a permission to publish two papers in one issue so that readers can learn in detail what the model is and how it operates. In this paper, we simply introduced the model to readers in the Introduction section (lines 73 to 82) and then delve into model calibration and application.**

**Thanks for the good suggestions to add clarification to the discussion. Four paragraphs (Revised manuscript: lines 286 to 316 and lines 369 to 378) were rewritten in the discussion section and other paragraphs had been revised to address these concerns.**

The manuscript constantly switches from decay to decomposition to decay. This is fairly confusing and unnecessary. I urge the authors to choose one term and stick with it. I prefer to call the process decomposition and the state it reaches decay. However, the authors can use either, but they should be consistent.

**Response: Thank you, “decomposition” is used now as suggested. However, in some cases, such as log decay class, we use the standard nomenclature to avoid confusion with the literature.**

Below are a number of specific comments that should be addressed to improve the clarity of the manuscript.

Specific comments (line)
14 This sentence has two problems. The first is the grammar is incorrect. It should use the word termed instead of term. But it is also factually incorrect. Although there is no set diameter cutoff, there is fine woody debris as well as coarse woody debris. Also woody debris contains woody materials such as bark was well as wood. This is not a good way to start an abstract.

**Response: Thank you. Reworded to “Coarse woody debris”.**

24 Is there some numerical indication of this degree of accuracy?

**Response: Thank you. We have added two sentences: The R^2^ (squared Pearson’s correlation coefficient) between the simulation and measurement was 0.80 for the model calibration and 0.82 for the model validation (P<0.01). The predicted mean mass loss from all logs was 5.4% lower than the measured mass loss and 1.4% lower than the calculated loss. (please see lines 25 to 28 of the revised manuscript).**

77 Although this is a review of another study, I have to remark that some of the factors mentioned are clearly confounded. Climate is related to location as is altitude. Hopefully these were not all used simultaneously.

**Response: Thank you. In the revision, “location” was deleted.**

79 I am not sure viable is the best word to use here. I suggest switching it to possible. I think the purpose of the current study is to test viability. The other study seems to suggest it is a possible model to be evaluated.

**Response: We revised “…CWDDAT is a viable tool…” to “…CWDDAT should be a potentially effective tool…” (lines 81 to 82 of the revised manuscript).**

81-83 I find the wording is odd in this section. I don’t see how model calibration affirms the design. Usually the first step in testing a model is to verify the various functions and responses to show they are as they should be. Perhaps this was taken care of in the earlier study. I guess one could then move on to whether the model can be calibrated to field data? I am not sure what mode availability is. Perhaps model availability? But even then I don’t know how “validating” indicates availability. I gather this was a corroboration phase in which one tests the model predictions to an independent data set. But it is worded in a most odd manner.

**Response:** **Yes. Function verification has been done in “Model Sensitivity analysis” in the first paper of the paired papers (“Coarse Woody Debris Decomposition Assessment Tool: Model development and sensitivity analysis” in this issue). Here “model calibration” using observations is only used to check to see if the model can work as same well as the function verification did. We revised the sentence (lines 83 to 86 of the revised manuscript).**

100 Is it possible to destroy an ecosystem short of a nuclear blast? Perhaps this could be reworded to be less value laden and more accurate? For example, I could see that a large share of the trees were felled by the storm. I can’t see that they were destroyed because then there would be no need to study the decomposition because the trees even in dead form would not be there.

**Response: Thank you. This paragraph has been revised (lines 106 to 114 of the revised manuscript). The sentence “Hurricane Hugo destroyed the forest in 1989” was revised to “Hurricane Hugo, a Category IV hurricane (1989), severely impacted the Santee EF. Over 80% of trees within WS80 were broken or uprooted, about 130 Mg ha^-1^ of CWD were left in the watershed, based on the post hurricane inventory”.**

121 I have concerns that only one disk was removed. The moisture content and extent of decay would likely vary with distance to the ends. Where was the disk removed from? A standard distance from the end? What was that? This is a critical detail.

**Response:** **Sorry for my mistake. We cut a disk from each edge of a FACE log, i.e. two disks were removed from a FACE log. We revised the sentence from “a disk was cut from each log” to “two disks (3-4 cm) were also cut from each end of the log, weighed in situ, and immediately placed in a plastic bag and sealed for transport to the laboratory” (lines 139 to 141 of the revised manuscript).**

**The disk was removed from each end of a FACE log, which is a potential issue as you note. We revised this paragraph (lines 294 to 306 of the revised manuscript). Because FACE wood decomposition experiment needs a long time period, we have to cut a disk from each end of a FACE log to keep the experiment continuous longer. If we want to have the middle disk, the expensive FACE logs will quickly become fragmented, and the long-term experiment would no longer be viable.**

128 This does not entirely seem correct. Validation as described seems to be what I would call verification. Validation or corroboration is a test of a model’s ability to predict against an independent data set. Maybe the authors are just following the terms laid out by Oreskes et al, but if that is the case I find the terms a bit of a muddle.

**Response: From the model developer side, since the model is not the real world, it may have some errors. In order to make sure the model is working correctly, model calibration and/or validation are a must, especially for a new model. This is why many journals do not publish any modeling work without calibration or validation. If a model failed to calibrate due to some errors, the code should be changed. However, our model passed the calibration using observations so that it was not necessary to modify the code. Since this model is new, we did both calibration and validation using different datasets to confirm the model availability, because different inputs may identify different model errors. Function verifications for this model have been done in model sensitivity analysis used a wide range of climate conditions from 14° to 65° N latitude and 58° – 139° W longitude (please see lines 73 to 82 of the revised manuscript).**

192 What I am not understanding is how one could possibly apply a non-linear model or a complex model to one data point in time. I think one is basically stuck with the linear negative exponential model if one has one point in time (or two if you count the initial value).

**Response: This is a power-exponential function with a moving point with time. It still has only one moving point in time. If we converted this model to logarithmic model, it would become a univariate polynomial. Of course, it is more complicated than a simple linear function or single exponential function.**

198 It would be helpful if this paragraph started with a topic sentence summarizing the results presented in the paragraph.

**Response: Thank you. A sentence was added, i.e., “Four metrics for evaluating model performance for the calibration step were used to determine if the model is in the line of the design objectives.” (revised manuscript, lines 219 to 220)**

205 It is not entirely clear what the numbers in the parentheses are referring to. Is this the fraction remaining? Or lost?

**Response:** **The values in the parentheses are the mass loss fraction. We have reworded “loss” to “loss fraction”. The sentence related to this has been revised to clarify (lines 225 to 227 of the revised version).**

212 I am not sure what to make of the statement “satisfies users’ basic expectations”. Wouldn’t that depend on the user and their expectations? It seems to imply some ability to read minds. Perhaps this could be reworded?

**Response: Thank you. We have reworded “satisfy users’ basic expectations” to “is applicable for assessing CWD decomposition across the range conditions on the EFs” (revised manuscript: lines 235 to 236).**
218 Again not sure what the numbers in the parentheses are referring to. Is this the total fraction mass lost?

**Response: Similarly, the values in the parentheses are the mass loss fraction so that we revised “loss” to “loss fraction”. The sentence related to this has also been revised to clarify (revised manuscript: lines 243 to 245).**

238 While this is a very interesting result and don’t question it, I do have to wonder about how the number was derived. Was this part of another study? Or if it is part of this study’s analysis: a few more details should be provided. For example, was the C concentration under logs monitored to determine the increase? How was the soil sampled? How was C concentration determined? To what depth? How was the change under logs scaled to the broadscale? Or was this a result of the models predictions?

**Response: This is from model prediction, so we reworded to “The predicted DOC…...,” (line 264 of the revised manuscript). We also revised the first sentence of this paragraph to “The CWDDAT also accounts for transfer of CWD-C into the forest floor and mineral soil” (revised manuscript: line 261).**

253 I am not following the logic here. It might be a matter of wording.

**Response: We have revised this sentence to “The predicted average wood mass loss from all samples, including those used to calibrate and validate the model, was 5.4% lower than the measured value, and 1.4% lower than the calculated mean, reflecting that the model slightly underestimates CWD decomposition rates.” (revised manuscript: lines 282 to 285).**

259 The details of disk location should have been in the methods. If one really wanted a good sample of conditions one should have removed disks at several locations so that a log level average could be derived.

**Response:** **Yes, the disk location has been mentioned in methods now (lines 139 to 141 of the revised manuscript).**

**As we mentioned above, we hope the decomposition experiment can continue for a time period as long as possible, so we only cut one disk from every end of each log per sampling. The FACE wood used for this experiment was in limited supply and no more exists for setting up additional studies.**

292-297 This seems more along the lines of results. Perhaps it needs to be set up better as a discussion point? Perhaps if it started “ A benefit of a mechanistic model is that it can provide insights into the basic processes causing decomposition”? Then go into the mechanisms that are suggested?

**Response: Thank you. The entire paragraph was moved to results, and has been revised (see lines 268 to 272 of the revised manuscript).**

504 A Y-intercept above 100% indicates the presence of a time-lag. Forcing the negative exponential through 100% artificially eliminates this time lag. A better method is to use a model that includes a time lag to get the intercept to be 100%. There are various models that can do this.

**Response: Thank you. We forced the intercept to 100%, which was only to show a small or large difference in the lag time. The actual function used to assess the decomposition rate is equation 7 in the manuscript. We revised the sentence (lines 332 to 333 of the revised manuscript).**

508 Was there any monitoring of the CWD mass in the watersheds? Or was there just an estimate of the initial input? Or was this just a prediction of the model that can’t be checked because the CWD was not repeatedly inventoried?

**Response: Biomass observation was conducted many times for this controlled watershed WS80 within the paired watershed system WS79 (see the revised manuscript: lines 106 to 108 and lines 110 to 113). However, the CWD on the watershed 80 used for this study was only based on the damage inventory after Hurricane Hugo (Hook et al., 1991; Haymond et al., 1996) (current version, lines 108 to 110).**

517 Did the logs contain any bark? Or were they all wood? Or was bark considered to be part of the wood? Was this the initial wood density or the average wood density over the course of the experiment? I suspect the former, but one can’t tell from what is written. I may have missed it, but I did not see any size 5 or 6 logs in the table. Why define size classes that were not used?

**Response: Thank you. The FACE logs were installed with intact bark. We added: “Those FACE logs used for this experiment had intact bark” (line 132).**

**The data in Table 2, including initial wood density, mass, size and species, are from the FACE logs that were used in our decomposition experiment. The sizes 5 and 6 were not used in the model calibration and validation because the size of the FACE logs is smaller than 30 cm in diameter (see Column “Diameter” in Table 2)**. **Although the sizes were not in the field experiment used to validate the model for the FWDE sites, they are sizes that the model can address, and the size 5 and 6 were used in model application for assessing CWD decay on WS80 (see Table 3).**

6. PLOS authors have the option to publish the peer review history of their article ([what does this mean?](https://journals.plos.org/plosone/s/editorial-and-peer-review-process#loc-peer-review-history)). If published, this will include your full peer review and any attached files.

If you choose “no”, your identity will remain anonymous but your review may still be made public.

**Do you want your identity to be public for this peer review?** For information about this choice, including consent withdrawal, please see our [Privacy Policy](https://www.plos.org/privacy-policy).

Reviewer #1: No

[NOTE: If reviewer comments were submitted as an attachment file, they will be attached to this email and accessible via the submission site. Please log into your account, locate the manuscript record, and check for the action link "View Attachments". If this link does not appear, there are no attachment files.]

While revising your submission, please upload your figure files to the Preflight Analysis and Conversion Engine (PACE) digital diagnostic tool, <https://pacev2.apexcovantage.com/>. PACE helps ensure that figures meet PLOS requirements. To use PACE, you must first register as a user. Registration is free. Then, login and navigate to the UPLOAD tab, where you will find detailed instructions on how to use the tool. If you encounter any issues or have any questions when using PACE, please email PLOS at [figures@plos.org](mailto:figures@plos.org). Please note that Supporting Information files do not need this step.

**Response: Thank you. We have done as you suggested. All figures have been uploaded to PACE for the analysis and correction. The figures in current version of manuscript are downloaded from PACE.**
